# Maintenance of the Shigella sonnei Virulence Plasmid Is Dependent on Its Repertoire and Amino Acid Sequence of Toxin-Antitoxin Systems

**DOI:** 10.1128/jb.00519-21

**Published:** 2022-03-15

**Authors:** Jessica E. Martyn, Giulia Pilla, Sarah Hollingshead, Kristoffer S. Winther, Susan Lea, Gareth McVicker, Christoph M. Tang

**Affiliations:** a Sir William Dunn School of Pathology, University of Oxford, United Kingdom; b Biologie des Bactéries Intracellulaires, Institut Pasteur, Paris, France; c Evotec, Abingdon, United Kingdom; d Biomolecular Sciences Department of Biology, Centre for Bacterial Stress Response and Persistence (BASP), University of Copenhagen, Copenhagen, Denmark; e Center for Structural Biology, Center for Cancer Research (CCR), National Cancer Institute at Frederick, Fort Detrick, MD, USA; f Antimicrobial Resistance, Omics and Microbiota Group, Department of Biosciences, Nottingham Trent University, Nottingham, United Kingdom; Duchossois Family Institute

**Keywords:** Shigella sonnei, virulence plasmid, TA systems, VapBC, insertion sequences, T3SS

## Abstract

Shigella sonnei is a major cause of bacillary dysentery and an increasing concern due to the spread of multidrug resistance. S. sonnei harbors pINV, an ∼210 kb plasmid that encodes a type III secretion system (T3SS), which is essential for virulence. During growth in the laboratory, avirulence arises spontaneously in S. sonnei at high frequency, hampering studies on and vaccine development against this important pathogen. Here, we investigated the molecular basis for the emergence of avirulence in S. sonnei and showed that avirulence mainly results from pINV loss, which is consistent with previous findings. Ancestral deletions have led to the loss from S. sonnei pINV of two toxin-antitoxin (TA) systems involved in plasmid maintenance, CcdAB and GmvAT, which are found on pINV in Shigella flexneri. We showed that the introduction of these TA systems into S. sonnei pINV reduced but did not eliminate pINV loss, while the single amino acid polymorphisms found in the S. sonnei VapBC TA system compared with S. flexneri VapBC also contributed to pINV loss. Avirulence also resulted from deletions of T3SS-associated genes in pINV through recombination between insertion sequences (ISs) on the plasmid. These events differed from those observed in S. flexneri due to the different distribution and repertoire of ISs. Our findings demonstrated that TA systems and ISs influenced plasmid dynamics and loss in S. sonnei and could be exploited for the design and evaluation of vaccines.

**IMPORTANCE** Shigella sonnei is the major cause of shigellosis in high-income and industrializing countries and is an emerging, multidrug-resistant pathogen. A significant challenge when studying this bacterium is that it spontaneously becomes avirulent during growth in the laboratory through loss of its virulence plasmid (pINV). Here, we deciphered the mechanisms leading to avirulence in S. sonnei and how the limited repertoire and amino acid sequences of plasmid-encoded toxin-antitoxin (TA) systems make the maintenance of pINV in this bacterium less efficient compared with Shigella flexneri. Our findings highlighted how subtle differences in plasmids in closely related species have marked effects and could be exploited to reduce plasmid loss in S. sonnei. This should facilitate research on this bacterium and vaccine development.

## INTRODUCTION

*Shigella* spp. are important human pathogens and the leading cause of bacillary dysentery, resulting in over 160,000 deaths annually (1). The Global Enteric Multicenter Study demonstrated that shigellosis is a major cause of moderate to severe diarrheal disease among children under five, with Shigella flexneri and Shigella sonnei accounting for around 90% of cases of shigellosis worldwide (2, 3). While S. flexneri causes endemic disease in low- and middle-income countries, S. sonnei dominates in wealthier countries where the bacterium is largely transmitted from person to person (4). Infection with S. sonnei has become a particular concern because of the emergence of antimicrobial-resistant strains (5, 6). Currently, there are no licensed vaccines against any *Shigella* spp.

All species of *Shigella* arose from Escherichia coli following the acquisition of an ∼210 kb plasmid, pINV (7), which encodes a type III secretion system (T3SS) on a 30 kb pathogenicity island (PAI) (8, 9). The T3SS delivers effector proteins, most of which are plasmid encoded, into host cells, leading to bacterial invasion and manipulation of host cell responses (10–13). Seminal studies demonstrated the contribution of pINV to *Shigella* virulence by showing that S. sonnei colonies, which lack pINV, cannot invade epithelial cells, a key step in *Shigella* virulence (14–16).

Avirulent S. sonnei cells arise at high frequency in the laboratory mostly due to pINV loss (14–17), posing a major obstacle for studying and developing vaccines against this species (18). For example, a significant proportion of strains in genome sequencing studies lack pINV (19), hampering phylogenetic analyses of plasmid-encoded virulence genes. In addition, pINV loss can confound studies of host-pathogen interactions and the development of animal models of infection (18). Furthermore, live attenuated and whole-cell S. sonnei vaccines can be noninvasive or lack pINV-encoded antigens if bacteria become avirulent (18, 20).

Here, we defined the molecular mechanisms responsible for the emergence of avirulence in S. sonnei. Consistent with previous work, we showed that loss of the S. sonnei virulence plasmid (pINV*^Ssonn^*) is the major event that results in avirulent S. sonnei (14, 15, 17). Previously, toxin-antitoxin (TA) systems were shown to be important for the maintenance of the S. flexneri virulence plasmid (pINV*^Sflex^*) by postsegregational killing (PSK) (17, 21). During PSK, if a daughter cell fails to inherit a plasmid, there is no *de novo* production of the antitoxin, which is preferentially degraded by cellular protease, allowing unopposed action of the toxin and resulting in growth arrest and/or death (22–24). In contrast to pINV*^Sflex^*, which has three functional TA systems (VapBC, GmvAT, and CcdAB) (17), we demonstrated that pINV*^Ssonn^* possessed two functional TA systems, RelBE and VapBC, which have the potential to promote pINV*^Ssonn^* maintenance through PSK. Our previous analysis revealed a 4.9 kb deletion that entirely encompasses *gmvAT* and a separate deletion that left a 23 bp fragment of *ccdA*, leading to the absence of both these TA systems from pINV*^Ssonn^* compared with pINV*^Sflex^* (17). Although introducing *ccdAB* and *gmvAT* into pINV*^Ssonn^* at sites corresponding to their location in pINV*^Sflex^* significantly reduced the emergence of avirulence, we found that pINV loss remained the dominant genetic event leading to avirulence. Furthermore, we showed that RelBE from pINV*^Ssonn^*, despite being functional, had no discernible effect on pINV*^Ssonn^* maintenance during growth in the laboratory. In contrast, although it was not possible to delete *vapBC* presumably due to its essential role in plasmid maintenance, we demonstrated that single amino acid substitutions in VapBC also contributed to pINV loss. Although we were unable to identify the mechanism(s) of how these polymorphisms contributed to plasmid maintenance, we showed that modification of this TA system can stabilize pINV*^Ssonn^*. Finally, extended deletions encompassing T3SS-associated genes could also lead to avirulence in S. sonnei. These intramolecular events resulted from recombination between homologous insertion sequences (ISs), which are abundant in *Shigella* (25). Intriguingly, IS-mediated deletions have been also detected in S. flexneri pINV*^Sflex^* (26) but were different from those observed in pINV*^Ssonn^* due to the distinct profile of ISs and TA systems on these plasmids. Understanding the mechanisms of plasmid maintenance and loss in S. sonnei should enable research on the biology of this important pathogen and be exploited for the development and evaluation of vaccines.

## RESULTS

### The absence of *gmv*AT and *ccd*AB from pINV*^Ssonn^* contributes to plasmid loss.

Spontaneous avirulence occurs more frequently in S. sonnei than S. flexneri due to pINV loss (14, 15, 17, 26, 27). pINV*^Sflex^* carries three functional plasmid maintenance TA systems, *vapBC*, *gmvAT*, and *ccdAB*, while *gmvAT* and *ccdAB* have been lost from pINV*^Ssonn^* through ancestral deletions (17). To determine whether the absence of *ccdAB* and *gmvAT* from pINV*^Ssonn^* is responsible for pINV loss, we analyzed a strain, *ccdAB*^+^/*gmvAT*^+^, with *ccdAB* and *gmvAT* and their promoters introduced into pINV*^Ssonn^* at sites corresponding to their positions in S. fexneri (17). The emergence of avirulent bacteria in *ccdAB*^+^/*gmvAT*^+^ and wild-type (WT) S. sonnei was assessed in Congo red (CR) binding assays. Avirulent *Shigella* that lost T3SS expression do not bind CR when grown on CR-containing media and appear white (CR^−^) while virulent T3SS-expressing bacteria bind CR and appear red (CR^+^) (28–30). Therefore, we measured the emergence of avirulence as the emergence of CR^−^ colonies and then characterized these colonies by multiplex PCR. To prevent pINV*^Ssonn^* loss before the start of experiments, we introduced a chloramphenicol resistance cassette (*cat*) into pINV in the WT strain and *ccdAB*^+^/*gmvAT*^+^ downstream of *vapC.* Bacteria were initially grown in the presence of the antibiotic then allowed to replicate in the absence of chloramphenicol for ∼25 generations by growth for 16 h at 37°C to allow for pINV loss during cell division. The emergence of avirulent colonies (CR^−^) was then quantified by plating bacteria to media containing CR.

**FIG 1:**
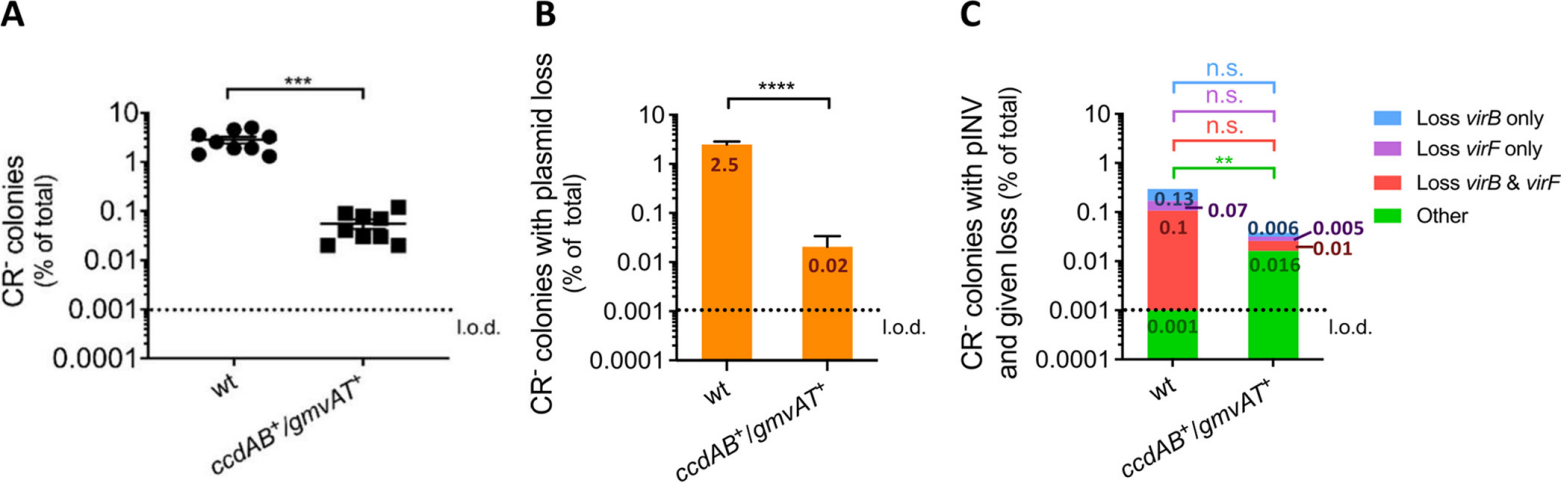
CR^−^ colonies of S. sonnei mostly result from loss of pINV. (A) The percentage of CR^−^ colonies emerging from wild-type S. sonnei 53G (WT) and a strain with *ccdAB* and *gmvAT* introduced into pINV (*ccdAB*^+^/*gmvAT*^+^) after growth at 37°C for ∼25 generations. Solid line: mean (*n* = 9 biological replicates). ***, *P ≤ *0.001 by parametric Welch’s *t* test. (B and C) The number of CR^−^ colonies possessing pINV but lacking specified plasmid genes were assayed by multiplex PCR and shown as a percentage of all colonies. Loss of the entire plasmid is inferred by loss of the origin of replication (B). “Other” refers to CR^−^ colonies that contain *virB*, *virF* and the origin of replication (C). The value of each category is indicated inside the corresponding bar portion. Eight independent CR^−^ colonies were obtained from each of nine biological replicates (i.e., 72 colonies were analyzed per strain). **, *P ≤ *0.01; ****; *P ≤ *0.0001; n.s., not significant; one-sample *t* test (parametric comparison of mean test/WT strain to 1) and Wilcoxon signed-rank test (nonparametric comparison of the median of strain/WT to 1, per specific event). l.o.d., limit of detection.

Consistent with previous work (17), the introduction of *gmvAT* and *ccdAB* into pINV*^Ssonn^* significantly reduced the emergence of avirulent CR^−^ colonies, by almost two orders of magnitude compared with the WT strain (*P* = 0.0003 for S. sonnei +/− *ccdAB*/*gmvAT*) (Fig. 1A) (17). To determine the molecular events leading to avirulence, we used multiplex PCR to examine CR^−^ bacteria for the presence of (i) *virF*, which is present on pINV but not in the T3SS PAI and encodes a transcriptional regulator of T3SS genes, (ii) *virB*, which is in the PAI and encodes a transcriptional regulator, (iii) *ori*, the pINV origin of replication which was used as a proxy for the presence of pINV (26, 31), and (iv) *hns* as a chromosomal control (Fig. 1B and C). pINV loss was the dominant cause of avirulence in the WT strain, accounting for over 99% of CR^−^ bacteria (Fig. 1B). Only 0.3% of avirulent bacteria emerging from wild-type S. sonnei retained pINV but had lost *virB* and/or *virF* (Fig. 1C). Introduction of *ccdAB* and *gmvAT* into pINV*^Ssonn^* significantly reduced pINV loss, which dropped from 2.5% to 0.02% of all colonies in the absence or presence of these TA systems, respectively (*P* < 0.0001) (Fig. 1B) and increased “other” events (*P* = 0.0070) (Fig. 1C), which lead to avirulence but not through the loss of *virB*, *virF*, and *ori*. This demonstrated that the introduction of *ccdAB* and *gmvAT* reduced pINV loss overall, making less frequent events more easily detectable. However, pINV loss was still the main genetic event leading to avirulence in *ccdAB*^+^/*gmvAT*^+^ and occurred in almost 50% of CR^−^ bacteria (Fig. 1B). Loss of *virB* and/or *virF*, were also detected at low frequency in both strains (Fig. 1C). Therefore, the ancestral loss of *ccdAB* and *gmvAT* from pINV*^Ssonn^* does not fully explain the high rate of pINV loss in S. sonnei.

### Polymorphisms in VapBC contribute to pINV loss in S. sonnei.

To further understand the factors responsible for the high rate of pINV loss in S. sonnei, we examined the sequence of the plasmid for TA systems. pINV*^Ssonn^* is predicted to encode a RelBE which is absent in pINV*^Sflex^* and a VapBC TA system (32). The RelBE TA system is encoded near the origin of replication and a closely related system stabilizes p307 in E. coli (see Fig. S1A and B in the supplemental material) (33). Analysis of whole-genome sequences from a global collection of 132 S. sonnei isolates (19) showed that pINV had been lost from most of the isolates; however, RelBE was found on pINV from all 43 S. sonnei isolates that contained the pINV sequence (Table S1). Although RelBE is a functional TA system, we found that it does not contribute to pINV maintenance under laboratory conditions (Fig. S1C and D).

Therefore, we focused our attention on the VapBC TA system, which is sufficient and necessary to maintain pINV*^Sflex^* and the only TA system found on pINV from both S. sonnei and S. flexneri (17). The VapC toxin cleaves the initiator tRNA, tRNA^fMet^, preventing initiation of translation, while the VapB antitoxin binds to and blocks the activity of VapC (34, 35). VapBC expression is autoregulated by conditional cooperativity through binding of heterocytomeric VapBC complex to two sites in the *vapBC* promoter, known as operator sites (*vapO*), resulting in transcriptional repression when VapB is in excess (34, 36). When pINV is lost following cell division, PSK occurs as VapB is readily degraded by Lon and cannot be replaced, leaving VapC free to cleave tRNA^fMet^ (35–37).

**FIG 2:**
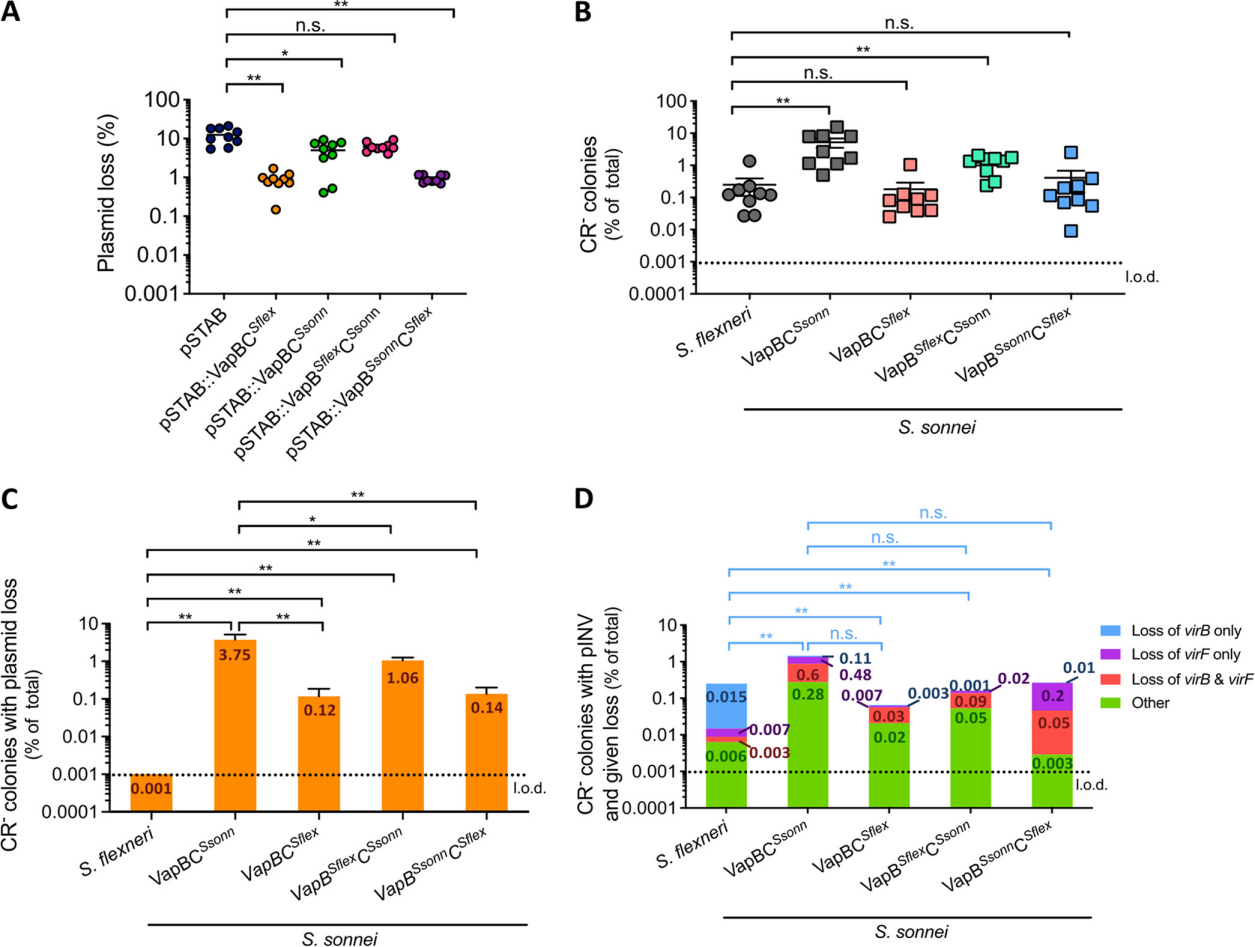
VapC polymorphisms affect plasmid maintenance. (A) The ability of VapBC*^Sflex^*, VapBC*^Ssonn^*, VapB*^Sflex^*C*^Ssonn^*, and VapB*^Ssonn^*C*^Sflex^* to maintain pSTAB in S. sonnei 53G lacking pINV. Each dot represents the result for a single colony and shows the percentage of all bacteria with plasmid loss. Solid line, mean + standard error of the mean (SEM) (*n* = 9 biological replicates). **, *P* ≤ 0.01; *, *P* ≤ 0.05; n.s. not significant; Wilcoxon signed-rank test (nonparametric comparison of median of VapBC+/empty pSTAB to 1). (B) The percentage of colonies that are CR^−^ of wild-type S. flexneri or S. sonnei (VapBC^Ssonn^), or S. sonnei with pINV harboring *vapBC^Sflex^* (VapBC*^Sflex^*), *vapB^Sflex^*C*^Ssonn^* (VapB*^Sflex^*C*^Ssonn^*) or *vapB^Ssonn^C^Sflex^* (VapB*^Ssonn^*C*^Sflex^*) after growth at 37°C for ∼25 generations; solid line, mean + SEM (*n* = 9 biological replicates). **, *P ≤ *0.01; n.s. not significant; Wilcoxon signed-rank test (nonparametric comparison of median of strain/S. flexneri to 1). (C and D) The number of CR^−^ colonies possessing pINV but lacking specified plasmid genes were assayed by multiplex PCR and shown as a percentage of all colonies. Loss of the entire plasmid is inferred by loss of the origin of replication (C). “Other” refers to CR^−^ colonies that contain *virB*, *virF*, and the origin of replication (D). The value of each category is indicated inside the corresponding bar portion. Eight independent CR^−^ colonies were obtained from each of three biological replicates (i.e., 72 colonies were analyzed per strain). ****, *P ≤ *0.0001; ***, *P ≤ *0.001; **, *P ≤ *0.01; *, *P ≤ *0.05; n.s. not significant; Wilcoxon signed-rank test (nonparametric comparison of the median of strain/S. flexneri or S. sonnei to 1, per specific event). l.o.d., limit of detection.

The promoter region of *vapBC*, including the *vapO* sites, was identical in S. flexneri and S. sonnei (Fig. S2A). Comparison of the predicted amino acid sequences of this TA system from S. flexneri M90T (accession number AL391753) and S. sonnei 53G (accession number NC_016833) revealed single amino acid differences in VapB and VapC (Fig. S2B and C). The polymorphic residue in VapB (T^58^A, S. flexneri versus S. sonnei) is located toward the C terminus of the antitoxin, which is involved in neutralizing VapC, while the polymorphic site in VapC (K^32^R, S. flexneri versus S. sonnei) is not located near its active site, in the self-dimerization interface, or its interface with VapB (Fig. S2B and C) (34). To determine if the *vapBC* sequences in S. flexneri M90T and S. sonnei 53G are representative of these species, we examined available S. flexneri and S. sonnei genome sequences for *vapBC*. The predicted amino acid sequence of VapBC in S. flexneri M90T and S. sonnei 53G were found in most isolates belonging to these species (83% and 78%, respectively) (Fig. 2SB and C). Therefore, we subsequently refer to VapB T^58^ as VapB*^Sflex^*, VapB A^58^ as VapB*^Ssonn^*, VapC K^32^ as VapC*^Sflex^*, and VapC R^32^ as VapC*^Ssonn^*.

We attempted to delete *vapBC* from pINV*^Ssonn^*. However, despite multiple attempts, we failed to obtain CR^+^ *vapBC* mutants, which is consistent with this TA system having a critical role in pINV*^Ssonn^* maintenance. Therefore, we analyzed the effect of the VapBC substitutions on plasmid maintenance using the model vector, pSTAB (37). pSTAB contains the origin of replication from pINV along with a *sacB*-*neo* cassette that allows detection of the presence or absence of the plasmid (37). We constructed pSTAB derivatives containing *vapBC* from S. sonnei (generating pSTAB::VapBC*^Ssonn^*) or from S. flexneri (pSTAB::VapBC*^Sflex^*), or containing chimeric *vapBC*s (i.e., pSTAB::VapB*^Sflex^*C*^Ssonn^* or pSTAB::VapB*^Ssonn^*C*^Sflex^*). The plasmids were introduced into S. sonnei 53G lacking pINV, and plasmid loss was determined after ∼25 generations by growth for 16 h at 37°C. While pSTAB with *vapBC^Sflex^* was lost at a substantially lower rate than empty pSTAB (*P* = 0.0039) (Fig. 2A), the introduction of *vapBC^Ssonn^* into pSTAB had a much more limited effect (*P* = 0.0195, pSTAB versus pSTAB::VapBC*^Ssonn^*) (Fig. 2A). pSTAB containing *vapB^Ssonn^C^Sflex^* exhibited significantly reduced plasmid loss compared with empty pSTAB, similar to pSTAB::VapBC*^Sflex^* (*P* = 0.0039, pSTAB versus pSTAB::VapB*^Ssonn^*C*^Sflex^*) (Fig. 2A). In contrast, *vapB^Sflex^vapC^Ssonn^* did not have a significant effect on pSTAB loss (*P* = 0.0742, pSTAB versus pSTAB::VapB*^Sflex^*C*^Ssonn^*) (Fig. 2A). These results indicate that the amino acid polymorphism in VapC (K^32^R) affects the function of the VapBC TA system on plasmid maintenance in pSTAB.

Next, we investigated whether the polymorphisms in S. sonnei VapBC affect pINV loss. We generated strains of S. sonnei containing pINV with S. sonnei *vapBC* (S. sonnei VapBC*^Ssonn^*), or with the locus replaced with *vapBC* from S. flexneri (S. sonnei VapBC*^Sflex^*), or a chimeric *vapBC* (i.e., S. sonnei VapB*^Sflex^*C*^Ssonn^* and S. sonnei VapB*^Ssonn^*C*^Sflex^*). A chloramphenicol resistance cassette (*cat*) was inserted downstream of *vapBC* in all strains to ensure retention of pINV before the start of experiments. As before, we quantified the emergence of CR^−^ colonies after ∼25 generations by growth for 16 h at 37°C in the absence of chloramphenicol. There was a significantly higher number of CR^−^ bacteria arising from S. sonnei VapBC*^Ssonn^* and S. sonnei VapB*^Sflex^*C*^Ssonn^* compared with wild-type S. flexneri (*P* = 0.0039) (Fig. 2B). In contrast, there was no difference in the number of CR^−^ bacteria emerging from S. sonnei VapBC*^Sflex^* or S. sonnei VapB*^Ssonn^*C*^Sflex^* compared with wild-type S. flexneri (*P* = 0.8203 and *P* = 0.4961) (Fig. 2B).

We also used multiplex PCR to investigate whether the single amino acid substitutions in VapBC altered the molecular events that lead to avirulence. Of note, the presence of *vapC* from S. flexneri led to a significant reduction in pINV loss as a cause of avirulence compared with S. sonnei *vapC* (S. sonnei VapBC*^Ssonn^* versus VapBC*^Sflex^*, *P* = 0.0039; S. sonnei VapBC*^Ssonn^* versus VapB*^Ssonn^*C*^Sflex^*, *P* = 0.0039) (Fig. 2C). In contrast, replacement of *vapB* from S. sonnei with *vapB* from S. flexneri had less effect on pINV loss (S. sonnei VapBC*^Ssonn^* versus VapB*^Sflex^*C*^Ssonn^*, *P* = 0.0391) (Fig. 2C). Therefore, the presence of S. flexneri *vapC* significantly decreased pINV loss, which was consistent with the results obtained with pSTAB. Furthermore, results demonstrated that, in addition to pINV loss, loss of *virB* and/or *virF* could also lead to avirulence in S. sonnei (Fig. 2D).

**FIG 3:**
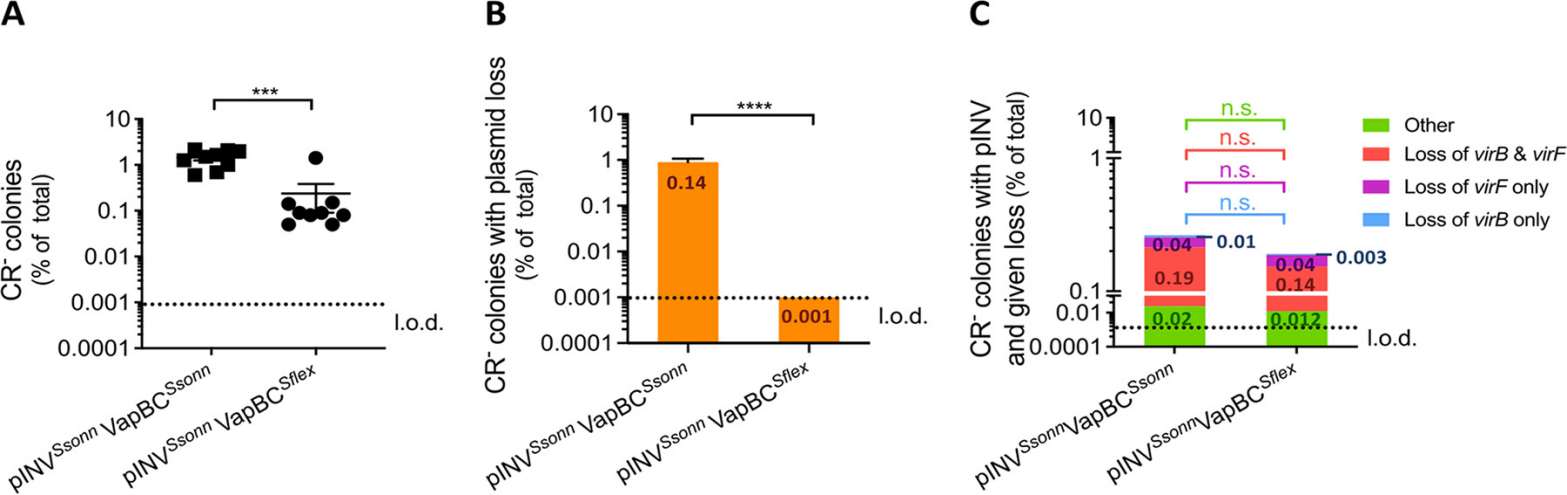
Loss of S. sonnei pINV is significantly reduced in a S. flexneri background. (A) The percentage of colonies that are CR^−^ of S. flexneri carrying pINV*^Ssonn^ vapBC^Ssonn^* or pINV*^Ssonn^ vapBC^Sflex^* after growth at 37°C for ∼25 generations; solid line, mean + S.E.M. (*n* = 9 biological replicates); ***, *P ≤ *0.001 by Mann-Whitney *t* test. (B and C) The number of CR^−^ colonies possessing pINV but lacking specified plasmid genes were assayed by multiplex PCR and shown as a percentage of all colonies. Loss of the entire plasmid is inferred by loss of the origin of replication (B); “other” refers to CR^−^ colonies that contain *virB*, *virF*, and the origin of replication (C). The value of each category is indicated inside the corresponding bar portion. Eight independent CR^−^ colonies were obtained from each of three biological replicates (i.e., 72 colonies were analyzed per strain). The value of each category is indicated inside the corresponding bar portion. ****, *P ≤ *0.0001; n.s. not significant; one-sample *t* test (parametric comparison of mean test/control strain to 1) and Wilcoxon signed-rank test (nonparametric comparison of the median of strain/control to 1, per specific event). l.o.d., limit of detection.

Because the genetic background can influence TA function (38), we also examined the effect of the VapBC polymorphisms in S. flexneri. We transferred derivatives of pINV*^Ssonn^* into S. flexneri lacking pINV by triparental mating. CR-binding assays with pINV*^Ssonn^* derivatives in S. flexneri demonstrated that the presence of *vapC^Sflex^* significantly reduced the emergence of CR^−^ colonies compared with *vapC^Ssonn^* (pINV*^Ssonn^* VapBC*^Ssonn^* versus pINV*^Ssonn^* VapBC*^Sflex^*, *P* = 0.0005) (Fig. 3A). Furthermore, multiplex PCR revealed that loss of the pINV was not detected among 72 CR^−^ colonies analyzed when *vapBC^Sflex^* was present on pINV*^Ssonn^* in S. flexneri (S. flexneri with pINV*^Ssonn^* VapBC*^Ssonn^* versus pINV*^Ssonn^* VapBC*^Sflex^*, *P* < 0.0001) (Fig. 3B and C).

Taken together, these results demonstrated that single amino acid substitutions in the VapBC TA system influenced the frequency of pINV loss in S. sonnei with the substitution in VapC (K32R) having a major effect. However, replacement of the *vapBC* in S. sonnei with *vapBC* from S. flexneri made pINV loss undetectable in S. flexneri, suggesting that the genetic background also influenced VapBC function in S. sonnei in addition to single amino acid substitutions.

### The effect of single amino acid substitutions on VapBC structure and function.

**FIG 4:**
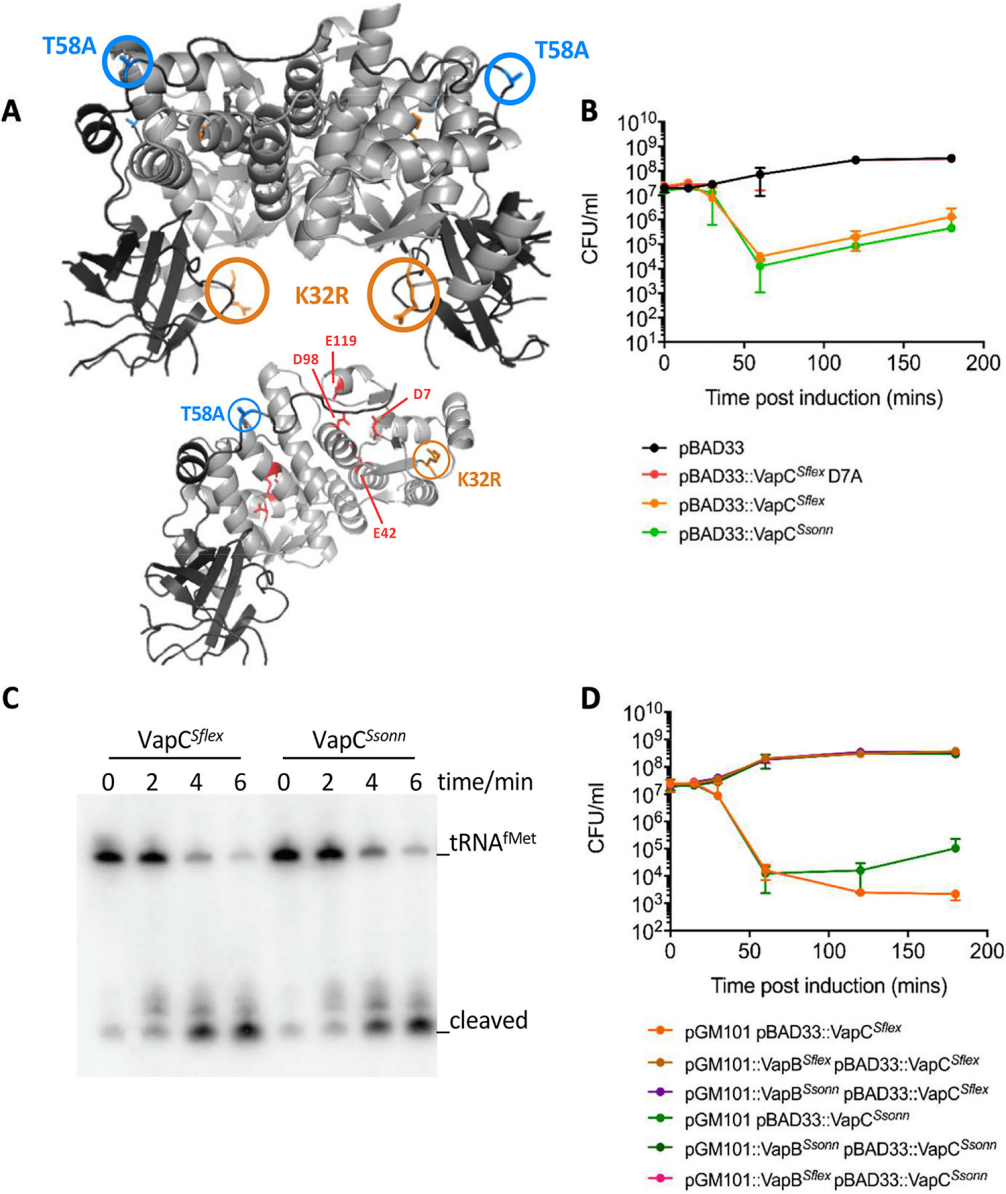
Analysis of the function of VapC polymorphisms. (A) Atomic structure of the S. sonnei 53G VapBC hetero-octamer (top) and the VapB*^Ssonn^* dimer in complex with a VapC*^Ssonn^* dimer (bottom). VapC*^Ssonn^* is in light gray, and VapB*^Ssonn^* is dark gray. The four residues comprising the VapC active site (D7, E42, D98, and E119) are highlighted in red. The location of the polymorphisms in VapB (T58A) and VapC (K32R) are highlighted in blue and orange, respectively. (B) Viability of S. sonnei 53G lacking pINV following expression VapC*^Ssonn^* or VapC*^Sflex^*, or a nonfunctional version of VapC*^Sflex^* (VapC*^Sflex^*D7A) from pBAD33. Empty pBAD33, control. (C) Northern blotting of VapC-mediated cleavage of tRNA^fMet^ in E. coli MG1655 at times (indicated) following expression of VapC*^Ssonn^* or VapC*^Sflex^* from an inducible promoter. (D) Viability of S. sonnei 53G lacking pINV following expression of VapC*^Ssonn^* or VapC*^Sflex^* from pBAD33 with VapB*^Ssonn^* or VapB*^Sflex^* under the expression of their promoters on pGM101. Error bars and standard deviation (SD).

Next, we investigated the mechanism(s) by which the amino acid substitutions in VapBC affect its function. VapBC forms a hetero-octameric complex that binds the operator sites in the promoter sequence (*vapO*) when VapB is in excess (34, 36). To establish whether the amino acid differences affect the overall architecture of the VapBC complex, we set up crystallization trials in the presence of *vapO*-containing DNA. However, the crystals obtained contained protein only, enabling us to determine the atomic structure of VapBC from S. sonnei to establish whether the amino acid differences affect the overall architecture of the VapBC complex (Fig. 4A; Table S2). Alignment of the S. sonnei and S. flexneri (PDB accession number 3TND) VapBC hetero-octamers demonstrated that they are highly similar (root mean square deviation of 0.460 Å over all Cα atoms) (Fig. 4A and Table S2), indicating that the substitutions did not significantly alter the complex.

To determine the influence of the VapC K^32^R polymorphism on its toxicity, we expressed each version of VapC under the control of an arabinose-inducible promoter in S. sonnei lacking pINV. An inactive version of the toxin, VapC D^7^A, was included as a negative-control (Fig. 4B) (35). Expression of *vapC^Sflex^* (encoding VapC K^32^) or *vapC^Ssonn^* (encoding VapC R^32^) led to a similar reduction in bacterial viability from 1 h postinduction onwards (*P* = 0.6867, one-way ANOVA, pBAD33::VapC*^Ssonn^* versus pBAD33::VapC*^Sflex^*) (Fig. 4B). Because the VapC K^32^R polymorphism was neither in the catalytic site nor at its interface with VapB, it could be involved in VapC cleavage of tRNA^fMet^. Therefore, to determine the influence of the VapC K^32^R polymorphism on VapC activity, we measured the cleavage of tRNA^fMet^, the target of the toxin, using an *in vivo* cleavage assay as previously described (35). VapC from S. sonnei or S. flexneri was expressed under an arabinose-inducible promoter in E. coli, and the cleavage of tRNA^fMet^ was followed by Northern blotting. There was no detectable difference in the cleavage of tRNA^fMet^ by VapC*^Ssonn^* and VapC*^Sflex^* (Fig. 4C).

We also investigated whether the VapC K^32^R substitution altered the capacity of either VapB*^Sflex^* or VapB*^Ssonn^* to act as an antitoxin, although this residue was not located at the TA interface. VapC was expressed under the control of an arabinose-inducible promoter in S. sonnei lacking pINV with either homologous or heterologous *vapB* coexpressed under the control of its native promoter. There was no significant difference in the ability of either VapB to neutralize either VapC (*P* = >0.9999, one-way ANOVA) (Fig. 4D).

In conclusion, we found that the polymorphisms in VapBC that influence pINV maintenance did not affect the structure of the VapBC complex, VapC toxicity, or the ability of VapB to act as an antitoxin.

### IS-mediated deletions in pINV*^Ssonn^* lead to avirulence.

pINV loss is not the only event that can result in avirulence in S. sonnei. Multiplex PCR of avirulent bacteria emerging from S. sonnei strains showed that some CR^−^ colonies retained pINV but lost *virB* and/or *virF* (Fig. 1C and 2D), which is similar to S. flexneri (26). Of note, there were marked differences in the frequency of these events in S. flexneri strains compared to S. sonnei strains (Fig. 2D). Loss of *virB* alone dominated in wild-type S. flexneri as previously shown (26) but was infrequently detected in S. sonnei (S. flexneri versus S. sonnei VapBC*^Ssonn^* and S. sonnei VapB*^Ssonn^*C*^Sflex^ P* = 0.0078; S. flexneri versus S. sonnei VapBC*^Sflex^* and S. sonnei VapB*^Sflex^*C*^Ssonn^*, *P* = 0.0039) (Fig. 2D). In contrast, loss of *virF* only and loss of *virF* with *virB* were rarely detected in S. flexneri but were relatively common in S. sonnei (Fig. 1C, 2D, and 3C).

**FIG 5:**
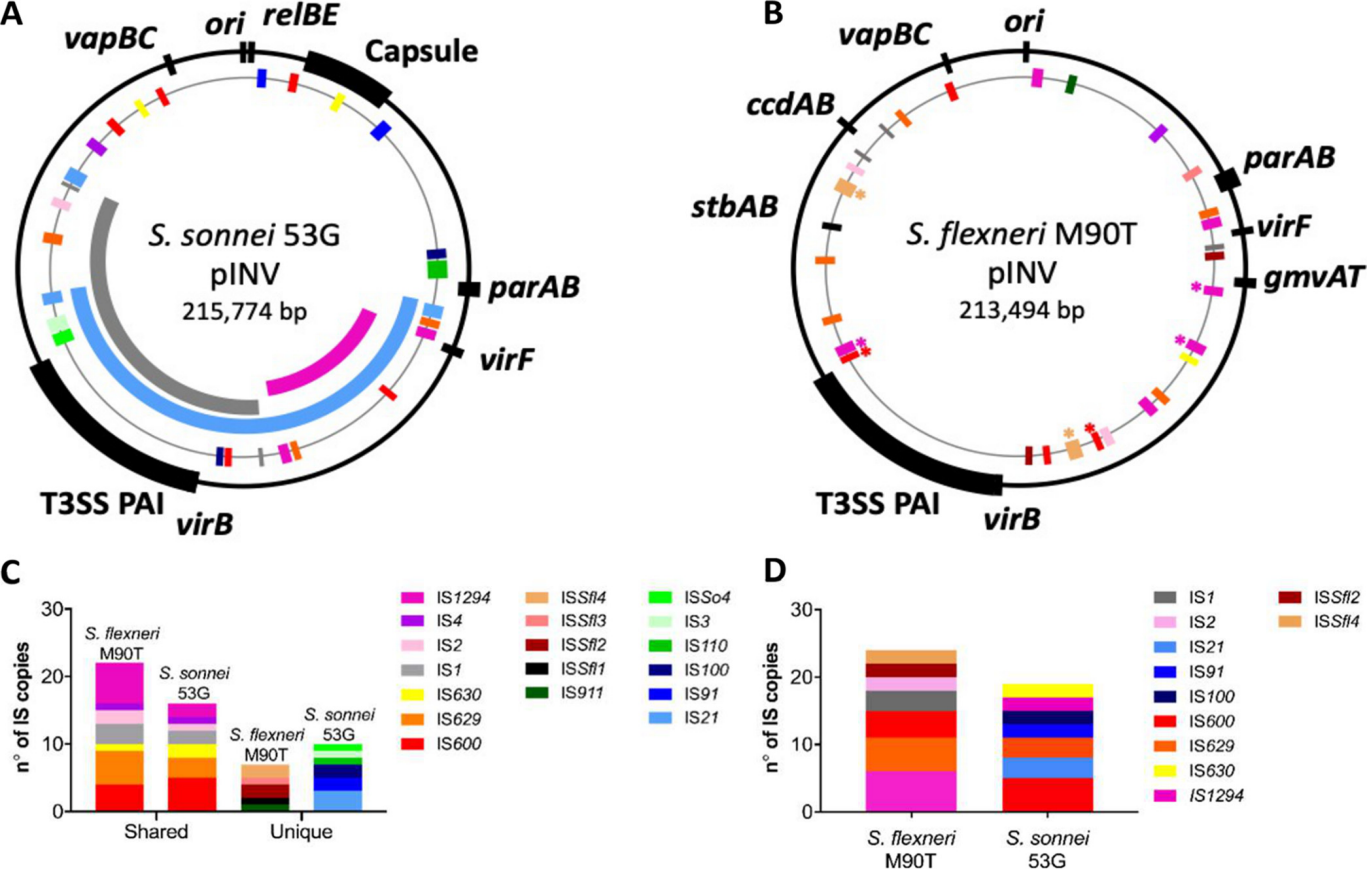
Distinct profile of ISs on pINV from S. flexneri 5a M90T and S. sonnei 53G dictate different deletions of the T3SS PAI-related genes. (A) Alignment of the three PAI deletions detected in S. sonnei 53G pINV (accession number NC_016833; indicated as the black outer ring). Insertion sequences are indicated as colored boxes and follow the same color code used in (B and C). (B) Schematic of the distribution of intact ISs on pINV from S. flexneri 5a M90T (accession number AL391753.1). ISs are colored as indicated. Asterisks indicate the ISs that mediate deletion of the T3SS PAI in S. flexneri (26). (C) Copies of ISs that are shared between the two strains or found in only one strain (unique). (D) ISs that are present in more than one copy.

Because the emergence of avirulence in S. flexneri usually occurs following recombination between homologous copies of ISs (26), we investigated whether the loss of *virB* and/or *virF* in S. sonnei resulted from IS-mediated deletions. We examined pINV*^Ssonn^* for pairs of ISs that occur in the same orientation and flank the T3SS PAI and/or *virF*. Using primers binding external to selected ISs, we amplified and sequenced potential IS-mediated deletions. This revealed that three pairs of ISs could contribute to avirulence in S. sonnei. Copies of IS*21* (positions 29959 to 32101 and 123799 to 125930 [blue arc in Fig. 5A]) mediated deletion of a 93.7 kb fragment and resulted in the loss of the T3SS PAI and *virF*. Copies of IS*1* (positions 73733 to 74500 and 146228 to 146996 [gray arc in Fig. 5A]) led to the loss of the T3SS PAI. Copies of IS*1294* (positions 33932 to 35621 and 68896 to 70585 [purple arc in Fig. 5A]) were involved in the deletion of *virF* alone.

To examine whether the deletions leading to avirulence in S. sonnei were due to the presence of distinct ISs on pINV compared with S. flexneri, we compared the content of ISs in pINV from these two species. In S. flexneri M90T pINV (pWR100), ISs accounted for 53% of open reading frames, and IS*Sfl4*, IS*1294*, and IS*600* were responsible for most T3SS PAI deletions (Fig. 5B) (26, 39). pINV*^Sflex^* harbored a total of 29 different ISs, consisting of 12 ISs types. IS*1294* was the most abundant IS (present in six copies) with five copies of IS*629* and four of IS*600* on the plasmid, respectively. Only four ISs were present in a single copy (Fig. 5C and D). In contrast, only ∼23% of open reading frames in pINV*^Ssonn^* were intact ISs with 26 ISs belonging to 13 different types. IS*600* was the most abundant IS in pINV*^Ssonn^* with five copies on the plasmid. The remaining ISs were present in up to three copies per plasmid, and seven were only present in a single copy (Fig. 5C and D). Therefore, there was a substantial difference in the repertoire and organization of ISs on the two plasmids with pINV*^Sflex^* harboring multiple copies of more ISs than pINV*^Ssonn^*.

Taken together, these results demonstrated that the number of ISs and their location influenced the different recombination events leading to avirulence in S. sonnei and S. flexneri.

## DISCUSSION

Here, we investigated the molecular mechanisms of pINV maintenance in S. sonnei and the reasons for the frequent emergence of avirulence (17). While three functional TA systems (VapBC, GmvAT, and CcdAB) are found on pINV*^Sflex^* (17, 21, 40, 41), pINV*^Sonn^* only has two TA systems, VapBC and RelBE, both of which are close to the origin of replication. While a RelBE homolog can maintain plasmids in E. coli (33, 42), this system does not contribute to the maintenance of pINV*^Ssonn^* under the conditions tested (see Fig. S1 in the supplemental material). Aside from the repertoire of TA systems, we showed that polymorphisms in VapBC and the number, type, and organization of ISs on pINV determine plasmid loss and plasticity in S. sonnei.

We confirmed that ancestral deletions affecting two TA systems, GmvAT and CcdAB, increase the loss of pINV*^Ssonn^* (17). However, even when GmvAT and CcdAB were introduced into pINV*^Ssonn^*, ∼50% of avirulent bacteria still resulted from pINV loss (Fig. 1B). VapBC (also referred to as MvpAT) is the only TA system present in pINV from both S. flexneri and S. sonnei and plays a critical role in maintaining pINV*^Sflex^* (17, 21, 40, 41). Given this, we hypothesized that differences in VapBC could also contribute to the high pINV loss in S. sonnei. We found that single amino acid polymorphisms in VapBC, particularly the substitution in VapC, have a marked effect on the function of this TA system in the model plasmid pSTAB and pINV.

The K^32^R substitution in VapC could affect pINV loss by several mechanisms during PSK. Of note, this change in VapC is not in the active site of the toxin, which is required for tRNA^fMet^ cleavage (35) or in the region that interacts with VapB or in the toxin dimerization region (Fig. 4A) (34, 36). To investigate how the VapC polymorphism influences pINV loss, we determined the atomic structure of the S. sonnei VapBC complex and found that the amino acid substitutions do not affect the overall structure of VapBC (Fig. 4A). Furthermore, there was no detectable change in the toxicity of VapC, its ability to cleave tRNA^fMet^, or its susceptibility to being neutralized by VapB (Fig. 4B to D). However, these assays might not be sufficiently sensitive to detect subtle yet important differences in VapC activity and/or VapB antitoxicity or interactions of VapBC with its operator DNA (Fig. S2A) (36). For PSK, VapC must be liberated from VapB. Therefore, it is also possible that the VapC K^32^ polymorphism reduces its affinity for VapB, increasing levels of free toxin in bacteria and enhancing PSK. Alternatively, the change in VapC might affect the degradation of VapB by the cellular proteases. Further studies are underway to examine these possibilities and the impact of the other VapBC sequence variants we identified (Fig. S2B and C) on the activity of this TA system.

ISs are critical for the architecture and evolution of plasmids in *Shigella* (39, 43). Indeed, recombination between ISs is likely to have resulted in the acquisition of the T3SS PAI (43). The spontaneous emergence of avirulence in S. flexneri is largely mediated by deletions of the T3SS PAI following intramolecular recombination between homologous copies of ISs (26, 31, 44). We found that some avirulent S. sonnei also arise following deletions of PAI-associated genes, but these events involve different ISs than S. flexneri because pINV from these two species harbor distinct IS profiles (Fig. 5C and D). Additionally, deletions involving the T3SS and its regulators may go undetected in S. sonnei because of the high level of pINV loss (Fig. 2C). As ISs can act as the substrate for horizontal gene flow, differences in ISs in pINV*^Ssonn^* and pINV*^Sflex^* might also affect the repertoire of T3SS effectors in the two species.

TA systems can stabilize local sequences by preventing IS-mediated deletions (26). Our results suggested that GmvAT might reduce IS*1294*-mediated loss of *virF* in S. flexneri because this system is located 7.4 kb from *virF* on pINV*^Sflex^* within a region bounded by two copies of IS*1294* (39). The absence of *gmvAT* from pINV*^Ssonn^* might explain our finding that avirulent strains lacking *virF* only were more common in S. sonnei than S. flexneri (Fig. 2D). This was further supported by the decrease in *virF* loss observed when we introduced *gmvAT* into its native site in pINV*^Ssonn^* (Fig. 1C).

S. sonnei is a highly successful pathogen despite having an unstable virulence plasmid when grown in the laboratory (14–16). pINV may be highly stable in S. sonnei *in vivo*, with environmental signals in the intestinal tract enhancing pINV maintenance. However, the emergence of avirulent bacteria does generate phenotypic heterogeneity, which might be beneficial for the survival of virulent and/or avirulent S. sonnei in certain circumstances. For example, rapidly replicating avirulent bacteria could act as an immune decoy in the intestinal tract or provoke local inflammatory responses and modify the local microenvironment in favor of virulent bacteria (45). Of note, pINV from S. sonnei harbors genes responsible for the biosynthesis of the O antigen which is incorporated into the lipopolysaccharide (LPS) and capsule of the bacterium (14, 15, 46, 47). Therefore, loss of pINV in S. sonnei also results in loss of the O antigen and capsule, which could enhance inflammatory responses to infection (47, 48).

Our findings could facilitate molecular studies of S. sonnei and aid the development of vaccines and animal/human challenge models (49). Vaccines based on live attenuated S. sonnei strains can be adversely influenced by pINV loss (18, 49–52), which might affect other vaccine strategies, including those based on outer membrane vesicles by reducing levels of T3SS effectors and O antigen, which are both critical immunogens (20). However, stabilization of pINV in live vaccine candidates would require attenuation of virulence through additional genetic changes (49). Finally, detailed knowledge of plasmid dynamics in S. sonnei might offer novel approaches to target the plasmid maintenance systems to combat the threat posed by this important human pathogen.

## MATERIALS AND METHODS

### Bacterial strains and growth.

Bacterial strains and plasmids used in this study are shown in Tables S3 and S4 in the supplemental material, respectively. *Shigella* spp. and E. coli were grown in tryptic soy broth (TSB; Sigma) and lysogeny broth (LB; Invitrogen), respectively, and 1.5% (wt/vol) agar (Oxoid) was added for solid media. Antibiotics were used at the following concentrations: carbenicillin, 100 μg/mL; chloramphenicol, 5 μg/mL (for S. flexneri) or 20 μg/mL (for S. sonnei); kanamycin, 50 μg/mL; streptomycin, 100 μg/mL. CR was added to TSB agar at a final concentration of 0.01% wt/vol (CR^−^ TSA). For sucrose selection, 1% (wt/vol) bacto-tryptone (Sigma), 0.5% (wt/vol), yeast extract (Sigma), and agar were autoclaved in water, and sucrose (VWR, a final concentration of 10%, wt/vol) added before pouring plates.

### Construction of strains.

Lambda Red recombination was employed to construct mutants (53). Approximately 1 kb of sequence upstream and downstream of the gene interest was used to flank an antibiotic resistance cassette. Fragments were amplified by PCR (primers used in this study are shown in Table S5) then ligated into pUC19 (54) using NEBuilder HiFi master mix (New England Biolabs). The resulting plasmids were transformed into E. coli DH5α and used as the template to generate approximately 1 μg of linear DNA by PCR, which was transformed by electroporation into S. sonnei 53G expressing the recombinase from pKD46 (53). Bacteria were plated onto CR-TSA plates containing appropriate antibiotics and incubated overnight at 37°C. Strains were checked by PCR and sequencing. For construction of S. sonnei *ccdAB*^+^/*gmvAT*^+^, VapBC*^Sson^*/WT, VapBC*^Sflex^*, VapB*^Sflex^*C*^Ssonn^*, VapB*^Ssonn^*C*^Sflxe^* and S. flexneri, the *cat* cassette was introduced at nucleotide 204,058 in S. sonnei 53G pINV (available at https://www.ncbi.nlm.nih.gov/nuccore/NC_016833.1?report=genbank) and at site 191,776 bp in S. flexneri M90T (accession number AL391753), respectively (26). For S. sonnei *ccdAB*^+^/*gmvAT*^+^, *ccdAB* and *gmvAT* were introduced into pINV*^Ssonn^* at sites corresponding to their positions in S. fexneri (17).

For the construction of pET28a-VapBC*^Ssonn^*, *vapB* and *vapC* were amplified from S. sonnei genomic DNA using the primers described in Table S5 and then inserted into pET28a digested with NdeI and XhoI using NEBuilder HiFi master mix.

### Triparental mating.

To transfer pINV*^Ssonn^* into S. flexneri, tri-parental mating was performed using S. sonnei 53G with a *cat* cassette downstream of *vapBC* (conferring chloramphenicol resistance) as the donor strain, S. flexneri BS176 (streptomycin resistant) as the recipient, and E. coli containing pRK2013 (conferring kanamycin resistance) as the helper strain (55, 56). Strains were grown separately overnight at 37°C in 10 mL LB liquid medium with appropriate antibiotics. Cultures were resuspended in 10 mL PBS after washing and then mixed at a ratio of 1:1:5 of donor, helper, and recipient, respectively, in 100 μL. Bacteria were spotted onto LB agar without antibiotics and incubated overnight at 37°C. Bacteria were harvested from spots and resuspended in 1 mL TSB and plated onto CR-TSA containing chloramphenicol and streptomycin to select for transconjugants.

### *sacB-neo* assays.

S. sonnei strains possessing pINV with the *sacB*-*neo* cassette in *mxiH* or pSTAB derivatives (17, 37) were grown from frozen stocks at 37°C for 16 h on solid LB medium to reach ∼25 generations. On three separate occasions, three colonies were resuspended in 100 μL PBS and serial dilutions plated on solid media with sucrose or with kanamycin only. PAI loss was calculated as the ratio of CFU on plates with sucrose and on plates without sucrose/with kanamycin and shown as a percentage.

### CR-binding assays.

*Shigella* spp. were grown at 37°C on CR-TSA plates containing chloramphenicol overnight to obtain single colonies. On three separate occasions, three independent CR^+^ colonies were resuspended in a 5 mL TSB liquid medium and incubated at 37°C with shaking at 180 rpm for 16 h (∼25 generations). Samples were diluted in PBS and plated onto CR-TSA and incubated overnight at 37°C before counting the number of CR^+^ and CR^−^ colonies. The proportion of CR^−^ colonies was quantified by dividing the number of emerging CR^−^ colonies by the total number of colonies (CR^+^ and CR^−^) and expressed as a percentage. Colonies were assessed by visual inspection.

### Multiplex PCR.

Multiplex PCR was used to detect *virF*, *virB*, and *ori* (the pINV origin of replication). *hns* was included as a chromosomal control (26). Reactions included *Taq* polymerase (Sigma-Aldrich) with an annealing temperature of 51.2°C and an extension time of 1.5 min. For each strain, eight CR^−^ colonies emerged from each biological repeat following the CR-binding assay were analyzed. For each strain, the percentage of all colonies showing a given gene loss was calculated by first quantifying the percentage of CR^−^ colonies with a particular gene loss and then adjusting the result using the percentage of CR^−^ colonies in the total bacterial population as described in the CR-binding assay.

### Toxicity, anti-toxicity, and VapC-mediated cleavage assays.

To assess the toxicity of VapC, the protein was expressed using pBAD33 (57) in S. sonnei 53G lacking pINV. Initially, bacteria were grown at 37°C with shaking at 180 rpm in LB liquid medium with 0.2% glucose (wt/vol) to repress toxin expression. At an optical density (OD) OD_600_ ∼0.1 cultures were pelleted by centrifugation at 3000 × *g* then resuspended in LB liquid medium with 0.2% arabinose (wt/vol) to induce toxin expression and grown at 37°C with shaking at 180 rpm. Aliquots of cultures were taken at time points following induction then serially diluted in PBS and plated onto LB solid media containing 0.2% glucose (wt/vol) to measure bacterial viability.

To assess the ability of VapB to prevent VapC toxicity, VapB from S. flexneri or S. sonnei was expressed from their native promoter on pGM101 in S. sonnei 53G lacking pINV with or without pBAD33::*vapC^Sflex^* or pBAD33::*vapC^Ssonn^* (17). Expression of VapC was induced as above in bacteria with or without a plasmid containing *vapB*, and viability was assessed by plating aliquots of cultures to solid media with 0.2% glucose (wt/vol).

To assess the ability of VapC to cleave tRNA^fMet^, E. coli MG1655 containing pBAD33::VapC*^Sflex^* or pBAD33:: VapC*^Ssonn^* were grown exponentially in LB liquid medium at 37°C with shaking at 180 rpm. At an OD_600_ of ∼0.4, toxin expression was induced by the addition of 0.2% arabinose (wt/vol). Samples (1 mL) were collected before (0 min) and 2, 4, and 6 min after addition of arabinose then immediately mixed with 125 μL of 5% phenol in ethanol on ice to prevent further RNA degradation. Samples were harvested by centrifugation at 3000 × *g* for 5 min at 4°C, and RNA was extracted using the hot phenol method as previously (58). Total RNA (2.5 μg) was denatured in formamide and separated on a denaturing 8% polyacrylamide gel (19:1) containing 8 M urea buffered in Tris-borate-EDTA (TBE). The RNA was transferred to a Zeta-Probe membrane (Bio-Rad) by electroblotting. Membranes were prehybridized in hybridization buffer (0.9 M NaCl, 0.05 M NaH_2_PO_4_, 0.05 M EDTA, 5× Denhardt’s solution [Thermo Fisher Scientific], 0.5% SDS and 550 μg salmon sperm DNA, pH 7.4) for 30 min at 42°C before the addition of the DNA probe. The probe was generated by 5′ phosphorylation of 30 pmol of tRNA^fMet^ specific DNA oligonucleotide (5′-CTTCGGGTTATGAGCCCGACGAGCTA) with 30 μCi [^32^P]-ATP using 1 μL T4 polynucleotide kinase (Thermo Fisher Scientific) in a total volume of 20 μL according to manufacturer’s instructions. Hybridization was continued overnight at 42°C. To reduce nonspecific hybridization, membranes were washed in 2×SSC (0.3 M NaCl, 0.03 M Na_3_C_6_H_5_O_7_) with 0.1% SDS at room temperature. Cleavage of tRNA^fMet^ was visualized by phosphorimaging.

### Protein purification and crystallography.

pET28a-VapBC*^Ssonn^* was transformed into E. coli C41. Following growth at 37°C to an OD_600_ of ∼0.8, expression was induced with 1 mM IPTG and cultures incubated for a further 3 h before harvesting by centrifugation at 5000 × *g* for 10 min. VapBC was purified as described previously (34). In brief, 150 mM VapBC*^Ssonn^* octamer was combined with double-stranded DNA (5′-ACAATAGATATACACAAGACATATCCAC-3′) resuspended in H_2_O using a ratio of 1:1.2 of VapBC*^Ssonn^* octamer to DNA. The VapBC*^Ssonn^*:DNA mixture was dialyzed in a solution of 25 mM Tris, pH 8.0, for 5 hours at room temperature in a Slide-A-Lyzer cassette (ThermoFisher Scientific) with a 3.5 kDa cutoff. Crystals were grown using the sitting drop method in 0.1 M ammonium sulfate, 0.3 M sodium formate, 0.1 M sodium cacodylate (pH 6.5), 3% γ-PGA, and 5% PEG 4000 at a ratio of 0.4:0.6 of protein to mother liquor. Crystals obtained were formed of VapBC*^Ssonn^* alone. Crystals were briefly incubated in a solution of crystallization buffer supplemented with 40% ethylene glycol followed by flash freezing in liquid nitrogen. Data were collected on beamline I04-1 at Diamond Light Source and indexed, scaled, and reduced using 2× dials (59) within ISPyB (60). The structure was solved by molecular replacement using PHASER (61) within CCP4i (62) with the structure of VapBC*^Sflex^* (accession number 3TND), (34) as the starting model. Iterative manual rebuilding and refinement using Coot (63) and PHENIX (64) led to the models described in Table S2.

### Bioinformatics.

Schematics of the position of RelBE or IS pairs on pINV from *Shigella* spp. were created from the sequence of S. flexneri M90T pINV (pWR100, accession number AL391753.1), and S. sonnei 53G pINV (accession number NC_016833.1). To determine the presence/absence of RelBE in a collection of S. sonnei from Holt et al. 2012 BLAST (BLASTn) v2.9.0 was used (19). Alignment of RelBE was performed using BLAST (BLASTn and BLASTp) v2.9.0 and subsequently Clustal O of sequence from RelBE from p307 from E. coli (accession number M26308) and S. sonnei 53G pINV (accession number NC_016833.1). Polymorphisms in VapB and VapC were identified in available S. flexneri or S. sonnei plasmid sequences using BLASTp (v2.9.0) and alignment with Clustal O (v1.2.4).

### Statistical analyses.

All data were analyzed using GraphPad Prism (version 7.4). The following statistical analyses were used as indicated in the legends of figures or the text: *t* test, one-sample *t* test/Wilcoxon signed-rank test, one-way ANOVA, and two-way ANOVA. For *t* test, data were first analyzed for normal distribution using the Shapiro-Wilk test, and, depending on the result, parametric (Welch’s *t* test) or nonparametric *t* test (Mann-Whitney test) were performed. For one-sample *t* test/Wilcoxon signed-rank test analysis, data were log-transformed and divided by the respective control strain. The Shapiro-Wilk test was then used to test for normal distribution. If data were normally distributed, a parametric one-sample *t* test was performed to compare the mean of data of each strain to 1 because 1 is the result of the ratio control/control or to the limit of the detection (LOD = 0.00001, i.e., 0.001%), which was equal to −5 log LOD in the absence of the control strain. If the data were not normally distributed, Wilcoxon signed-rank test was performed, comparing the median of data of each strain to 1 or the LOD. For one-way ANOVA, Kruskal-Wallis, Dunn’s multiple-comparison test was performed. For two-way ANOVA, Sidak’s or Tukey’s multiple-comparison test was performed.

### Data availability.

 Coordinates and structure factors have been deposited in the Protein Data Bank (accession number 6SD6).
