# Supplementary material for: Maintenance of the Shigella sonnei Virulence Plasmid Is Dependent on Its Repertoire and Amino Acid Sequence of Toxin-Antitoxin Systems
Source: J Bacteriol. 2022 Mar 15;204(3):e00519-21. doi: 10.1128/jb.00519-21 (PMC8923223; doi:10.1128/jb.00519-21)
Supplement: Supplemental file 1 — Fig. S1 and S2 and Tables S1 to S5. Download jb.00519-21-s0001.pdf, PDF file, 0.9 MB [file jb.00519-21-s0001.pdf]

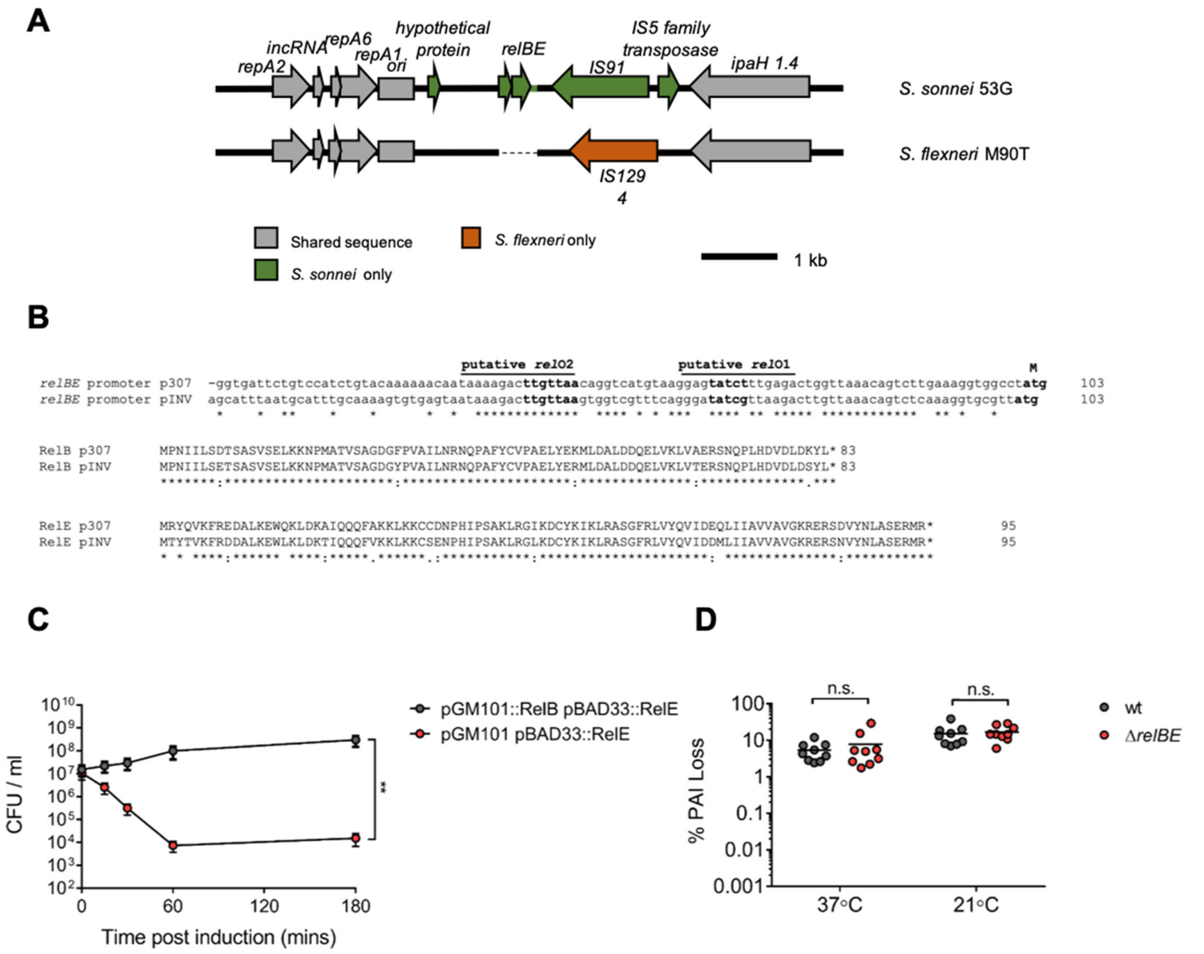

Figure S1. ***S. sonnei*** **RelBE is a functional TA system that does not contribute to pINV^{Ssonn} stability.** **(A)** Comparison of *S. flexneri* M90T 5a pINV (AL391753) and *S. sonnei* 53G pINV (NC_016833) near the origin of replication (*ori*). **(B)** Alignment of the promoter region of *relBE* and its amino acid sequence from *E. coli* p307 (M26308) and from *S. sonnei* pINV (NC_016833) by Clustal O (1.2.4). The predicted -10 and -35 are highlighted in bold. The putative *relO* sites are highlighted. The first codon of RelB is indicated using a M. Asterisks indicate identical nucleotides/amino acids, colons show amino acids with similar properties, and periods indicate amino acids sharing weakly similar properties. **(C)** Viability of *S. sonnei* 53G lacking pINV following expression of *relE* from pBAD33 in the presence/absence of *relB* on pGM101. **, $p < 0.01$ by two-way ANOVA with Sidak's multiple comparisons test (n = 3 independent experiments, error bars show S.D.). **(D)** Loss of the PAI measured using *sacB-neo* on pINV^{Ssonn} with or without *relBE* following growth at 37°C or 21°C for ~ 25 generations. n.s. not significant by Mann-Whitney t-test (n = 9 colonies from three independent experiments).

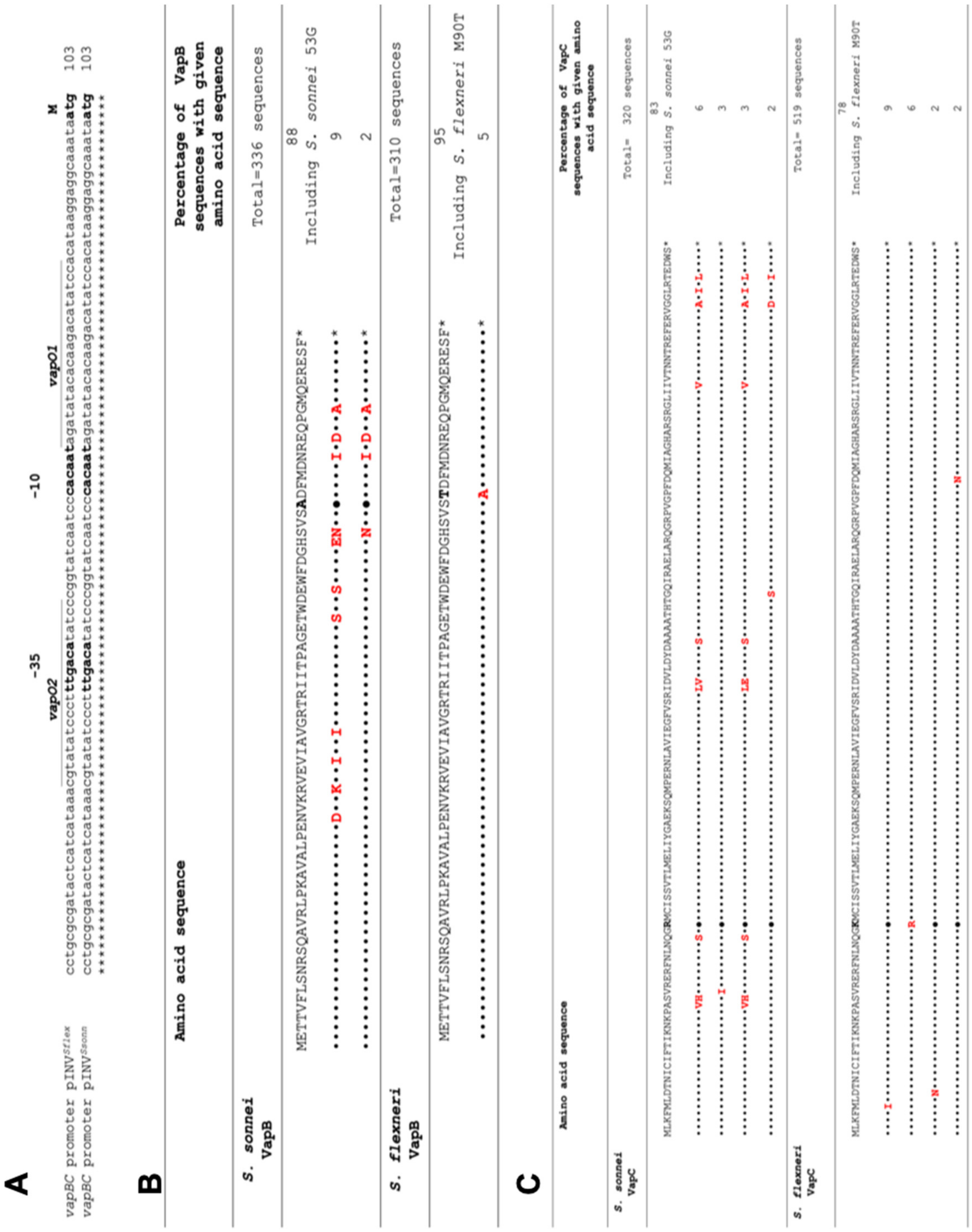

Figure S2. Polymorphisms in VapBC from *Shigella* spp.

(A) Alignment of the *vapBC* promoter from *S. sonnei* and *S. flexneri* by CLUSTAL O (1.2.4). The predicted -10 and -35 sequences are highlighted in bold. The putative *vapO* sites are indicated. Asterisks indicate identical nucleotides. **(B and C)** Comparison of the predicted amino acid sequences of *S. sonnei* and *S. flexneri* VapB and VapC. Polymorphic residues are indicated in red with identical amino acids shown as circles. The single polymorphic differences between the *S. flexneri* and *S. sonnei* versions of VapB and VapC (T58A and K32R, respectively) are indicated in bold. Reference strains are *S. flexneri* 5a M90T pINV (AL391753) and *S. sonnei* 53G pINV (NC_016833).

Table S1: The presence of *relBE* on pINV^{Ssonn}

Sequence ID Holt *et al.*	Accession number	*relBE*	ori	*vapBC*
Sh74369	SRA: ERX2006371	present	present	present
CS1	NZ_CXFA00000000	present	-	present
20071599	NZ_CXET00000000	present	present	present
Sh9810267	NZ_CXDR00000000	present	present	present
ShIB716	SRA:ERX2005632	present	present	present
ShIB717	NZ_CXBM00000000	present	present	present
ShIB748	NZ_CXBQ00000000	present	present	present
ShIB697	NZ_CXBP00000000	present	present	present
Sh2073	SRA: ERX2005582	present	present	present
CS8	NZ_CXEZ00000000	-	-	-
ShIB3277	SRA: ERX2005656	present	present	present
ShIB3580	NZ_CXBZ00000000	present	present	present
ShIB3599	NZ_CXCP00000000	present	present	present
ShIB3488	NZ_CXBW00000000	present	present	present
ShIB3507	NZ_CXBT00000000	present	present	present
CS7	NZ_CXEY00000000	present	present	present
20010007	NZ_CXEX00000000	present	present	present
20062313	NZ_CXEW00000000	present	present	present
ShIB3300	NZ_CXCL00000000	present	present	present
CS20	NZ_CXEE00000000	present	present	present
CS14	NZ_CXEI00000000	present	present	present
19904011	NZ_CXES00000000	present	present	present
Sh970044	SRA: ERS009863	present	present	present
20011685	NZ_CXER00000000	present	present	present
20031275	NZ_CXEM00000000	present	present	present
Sh60108	NZ_CXAS00000000	present	present	present
20062087	NZ_CXEU00000000	present	present	present
ShIB691	NZ_CXAZ00000000	present	present	present
20040924	NZ_CXEV00000000	present	present	present
ShIB3374	SRA: ERS009826	present	present	present
20021122	NZ_CXEG00000000	present	present	present
ShIB2013	NZ_CXBN00000000	present	present	present
ShIB2012	SRA:ERX2005602	present	present	present
ShIB1976	SRA: ERS009804	present	present	present
ShIB1980	NZ_CXBI00000000	present	present	present
ShIB1985	NZ_CXCG00000000	present	present	present
ShIB2015	NZ_CXCF00000000	present	present	present
ShIB2008	NZ_CXBV00000000	present	present	present
ShIB2000	NZ_CXBY00000000	present	present	present
ShIB1970	NZ_CXBL00000000	present	present	present

ShIB2004	NZ_CXCD00000000	present	present	present
ShIB2024	NZ_CXCA00000000	present	present	present
ShIB2018	NZ_CXBU00000000	present	present	present
ShIB1993	NZ_CXBO00000000	present	present	present
ShIB1990	NZ_CXCK00000000	present	present	present
ShIB1987	NZ_CXBR00000000	present	present	present

Table S2. Data collection and refinement statistics for VapBC^{Ssonn}

	VapBC (6SD6)
Wavelength	
Resolution range	35.15 - 2.61 (2.703 - 2.61)
Space group	P 32 2 1
Unit cell	95.8732 95.8732 116.389 90 90 120
Total reflections	38544 (3790)
Unique reflections	19274 (1895)
Multiplicity	2.0 (2.0)
Completeness (%)	99.06 (97.32)
Mean I/sigma(I)	9.82 (0.89)
Wilson B-factor	71.32
R-merge	0.0358 (0.9819)
R-meas	0.05062 (1.389)
R-pim	0.0358 (0.9819)
CC1/2	0.999 (0.489)
CC*	1 (0.811)
Reflections used in refinement	19114 (1854)
Reflections used for R-free	931 (85)
R-work	0.2063 (0.3485)
R-free	0.2359 (0.4159)
CC(work)	0.964 (0.615)
CC(free)	0.988 (0.559)
Number of non-hydrogen atoms	3191
macromolecules	3164
solvent	27
Protein residues	402
RMS(bonds)	0.002
RMS(angles)	0.47
Ramachandran favored (%)	96.70
Ramachandran allowed (%)	3.30
Ramachandran outliers (%)	0.00
Rotamer outliers (%)	4.41
Clashscore	3.00
Average B-factor	80.58
macromolecules	80.65
solvent	72.81

Statistics for the highest-resolution shell are shown in parentheses.

Table S3: Bacterial strains used in this study

Strain name	Relevant genotype/description	Reference
S. flexneri M90T	Wild-type *S. flexneri* serotype 5a	Zychlinsky *et al.* 1992
BS176	*S. flexneri* M90T lacking pINV	Zychlinsky *et al.* 1992
S. sonnei 53G	Wild-type *S. sonnei*	Kopecko *et al.* 1980
S. flexneri vapBC-cat	*S. flexneri* with *cat* downstream of *vapBC*	Pilla *et al.*, 2017
S. sonnei vapBC-cat	*S. sonnei* with *cat* downstream of *vapBC*	This study
S. sonnei $pINV^-$	*S. sonnei* lacking pINV	McVicker & Tang, 2016
S. sonnei Δ*relBE*	*S. sonnei* Δ*relBE mxiH::sacB-neo*	This study
S. sonnei $ccdAB^+/gmvAT^+$	*S. sonnei* containing *ccdAB-cat gmvAT*	McVicker & Tang, 2016
S. sonnei $VapBC^{Sflex}$	*S. sonnei* containing $vapBC^{Sflex}$ with *cat* downstream	This study
S. sonnei $VapB^{Ssonn}C^{Sflex}$	*S. sonnei* containing $vapB^{Ssonn}C^{Sflex}$ with *cat* downstream	This study
S. sonnei $VapB^{Sflex}C^{Ssonn}$	*S. sonnei* containing $vapB^{Sflex}C^{Ssonn}$ with *cat* downstream	This study
BS176 $pINV^{Ssonn}$ $VapBC^{Ssonn}$	BS176 containing $pINV^{Ssonn}$ $VapBC^{Ssonn}$ with *cat* downstream	This study
BS176 $pINV^{Ssonn}$ $VapBC^{Sflex}$	BS176 containing $pINV^{Ssonn}$ $VapBC^{Sflex}$ with *cat* downstream	This study
E. coli DH5α	*fhuA*2 Δ(*argF-lacZ*) *U169 phoA glnV44 Φ80* Δ(*lacZ*)*M15 gyrA96 recA1 relA1 endA1 thi-1 hsdR17*	Hanahan 1983
E. coli MG1655	Wild-type *E. coli* K-12	Guyer *et al.* 1981
E. coli C41(DE3)	*ompT hsdSB* (*rB- mB-*) *gal dcm* (DE3)	Miroux & Walker, 1996

Table S4: Plasmids used in this study

Plasmid	Relevant genotype or description	Antibiotic resistance	Reference
pUC19	Cloning vector	Amp^r	Yanisch-Perron *et al.* 1985
pUC19-*relBE*::*cat*	Cloning vector to introduce *relBE* with a downstream *cat* cassette into *S. sonnei*	Amp^r	This study
pUC19-*vapBC::cat*	Cloning vector to introduce *vapBC* with a downstream *cat* cassette into *S. sonnei*	Amp^r	This study
pUC19-*vapBC^{Sflex}*	Cloning vector to introduce *vapBC* from *S. flexneri* with a downstream *cat* cassette into *S. sonnei*	Amp^r	This study
pUC19-*vapB^{Ssonn}C^{Sflex}*	to introduce *vapC* from *S. flexneri* into *S. sonnei vapBC-cat*	Amp^r	This study
pUC19-*vapB^{Sflex}C^{Ssonn}*	Cloning vector to introduce *vapB* from *S. flexneri* into *S. sonnei vapBC-cat*	Amp^r	This study
pKD46	Plasmid expressing the λ red recombinase	Amp^r	Datsenko & Wanner, 2000
pCP20	Temperature sensitive plasmid encoding FLP recombinase	Amp^r	Datsenko & Wanner, 2000
pKD3	Plasmid with FRT-sites flanking a *cat* cassette	Kan^r	Datsenko & Wanner, 2000
pSTAB	6.3 kb plasmid containing the pINV ori and *sacB-neo*	Kan^r	McVicker *et al.* 2019
pSTAB::VapBC^{Sflex}	pSTAB with *S. flexneri vapBC*	Kan^r	This study
pSTAB::VapBC^{Ssonn}	pSTAB with *S. sonnei vapBC*	Kan^r	This study
pSTAB::VapB^{Ssonn}C^{Sflex}	pSTAB with *vapB* from *S. sonnei* and *vapC* from *S. flexneri*	Kan^r	This study
pSTAB::VapB^{Sflex}C^{Ssonn}	pSTAB with *vapB* from *S. flexneri* and *vapC* from *S.sonnei*	Kan^r	This study
pBAD33	Empty vector	Cam^r	Guzman *et al.* 1995
pBAD33::RelE	pBAD33 with *relE* under an arabinose-inducible promoter	Cam^r	This study
pBAD33::VapC^{Sflex}	pBAD33 with *S. flexneri vapC* under an arabinose-inducible promoter	Cam^r	McVicker & Tang, 2016
pBAD33::VapC^{Sflex} D7A	pBAD33 with *S. flexneri vapC*^{D7A} under an arabinose-inducible promoter	Cam^r	McVicker & Tang, 2016
pBAD33::VapC^{Ssonn}	pBAD33 with *S. sonnei vapC* under an arabinose-inducible promoter	Cam^r	This study
pGM101	Vector compatible with pBAD33	Amp^r	McVicker & Tang, 2016
pGM101::RelB	pGM101 with *relB* under its native promoter		This study
pGM101::VapB^{Sflex}	pGM101 with *vapB* from *S. flexneri* under its native promoter	Amp^r	McVicker & Tang, 2016
pGM101::VapB^{Ssonn}	pGM101 with *vapC* from *S. sonnei* under its native promoter	Amp^r	This study
pINV^{Ssonn} VapBC^{Ssonn}	*S. sonnei* pINV with *vapBC^{Ssonn}-cat*	Cam^r	This study
pINV^{Ssonn} VapBC^{Sflex}	*S. sonnei* pINV with *vapBC^{Sflex}-cat*	Cam^r	This study
pET28a-VapBC^{Ssonn}	pET28a with N-terminal His tagged VapC and untagged VapB from *S. sonnei*	Kan^r	This study

Table S5: Primers used in this study

Primer	Sequence (5' - 3')	Purpose
GM371	TGGGCTAGCGAATTCGAGCTCATGATGTGGACCTGGAC	Construction of pBAD33-*relE*
GM372	CTCATCCGCCAAAACAGCCAAGCTTCTATCTCATTCTCTCACTGG	Construction of pBAD33-*relE*
GM373	CGCGAAGGCGAAGCGGCATGCGCTGAAAACGGGTATGGC	Construction of pGM101-*relB*
GM374	CTCATCCGCCAAAACAGCCAAGCTTCATAAATAACTGTCCAGGTCC	Construction of pGM101-*relB*
GM399	AAACGACGGCCAGTGAATTCGAGCTCAAGTGACCTCCTCAGAATAATC	Construction of pUC19-*relBE::cat*
GM400	CTTCGAAGCAGCTCCAGCCTACACATTAACAAGTCTTTATTACTCACAC	Construction of pUC19-*relBE::cat*
GM401	CATATGGACCATGGCTAATTCCCATTAACATATAAATAACCTTTCCCG	Construction of pUC19-*relBE::cat*
GM402	AACAGCTATGACCATGATTACGCCAAGCTTGTACATGTGTCTGTAAC	Construction of pUC19-*relBE::cat*
GP007	ACGGCCAGTGAATTCGAGCTCGTGAAGCGGGTCCGGGTG	Construction of *S. sonnei vapBC-cat, S. sonnei* vapBC^{Sflex}, *S. sonnei* vapB^{Ssonn}C^{Sflex}, *S. sonnei* vapB^{Sflex}C^{Ssonn}
GP010	CCATGGCTAATTCCCATTCAGCTCCAGTCTTCAGTTC	Construction of *S. sonnei vapBC-cat, S. sonnei* VapBC^{Sflex}, *S. sonnei* vapB^{Ssonn}C^{Sflex}, *S. sonnei* vapB^{Sflex}C^{Ssonn}
GP011	AGCCTACACACCTGTTCATCAGAAATCATCTC	Construction of *S. sonnei vapBC-cat, S. sonnei* vapBC^{Sflex}, *S. sonnei* vapB^{Ssonn}C^{Sflex}, *S. sonnei* vapB^{Sflex}C^{Ssonn}
GP141	CATGATTACGCCAAGCTGTAATCGTCGTGTGTGG	Construction of *S. sonnei vapBC-cat, S. sonnei* vapBC^{Sflex}, *S. sonnei* vapB^{Ssonn}C^{Sflex}, *S. sonnei* vapB^{Sflex}C^{Ssonn}
GM174	TGTGTAGGCTGGAGCTGCTT	PCR FRT flanked *cat* cassette from pKD3
GM175	ATGGGAATTAGCCATGGTCC	PCR FRT flanked *cat* cassette from pKD3
GM393	CCATATGGCTAGCATGAGCGGATCCGTAATTATCCTTGTCGGTATAATAATTACC	Construction of pSTAB-VapBC plasmids
GM394	GGTGCTCGAGTGCGGCCGCAAGCTTGAACAGGTCAGCTCCAG	Construction of pSTAB-VapBC plasmids
GM105	AGGCGAAGCGGCATGCGATGCCTGTTACTGCCA	pGM101-*vapB^{Ssonn}*, pGM101-*vapB^{Sflex}*
GM106	CCGCCAAAACAGCCATCAGAATGACTCCCTTTCC	pGM101-*vapB^{Ssonn}*, pGM101-*vapB^{Sflex}*
GM122	CTAGCGAATTCGAGCTCATGCAGGAAAGGGAGTC	pBAD33-*vapC^{Ssonn}*, pBAD33-*vapC^{Sflex}*
GM123	CCGCCAAAACAGCCAAGCTTCAGCTCCAGTCTTCAGTTC	pBAD33-*vapC^{Ssonn}*, pBAD33-*vapC^{Sflex}*
GP99	CCTATCGATGGACATGAATGCAGG	Check of IS*21*-mediated deletion of T3SS-related genes
GP100	TCGCCTGTTTCACCGTATCTTC	Check of IS*21*-mediated deletion T3SS-related genes
GP145	GGAGCGGTATCAGGCAAACG	Check of IS*1294*-mediated deletion T3SS-related genes
GP146	GCTGACAGTGGTAATACGTTG	Check of IS*1294*-mediated deletion T3SS-related genes
GP147	CCGACCATATCCGCCATTG	Check of IS*1*-mediated deletion T3SS-related genes

GP148	CGTTTGCCTGATACCGCTCC	Check of IS*1*-mediated deletion T3SS-related genes
*hns*_F	TTGCAAAGGCGTTGAATTA	Multiplex PCR
*hns*_R	TTGCAAAGGCGTTGAATTA	Multiplex PCR
*virB*_F	ACATCAGAGCTCCACAAGAA	Multiplex PCR
*virB*_R	AGACGATAGATGGCGAGAAA	Multiplex PCR
*virF*_F	CTTAGCTTGTTGCACAGAGA	Multiplex PCR
*virF*_R	AAGATGGGCTTGATATTCCG	Multiplex PCR
*ori*_F	GTGACCTCCTCAGAATAATCC	Multiplex PCR
*ori*_R	AAAAGATACATTGCACCCTGT	Multiplex PCR
SH10	CTGGTGCCGCGCGGCAGCCATATGCTGAAGTTTATCGTCG	Construction of pET28a-$vapCB^{Ssonn}$
SH11	CCATTATTTGCCTCCTTATCAGCTCCAGTCTTCAGTTC	Construction of pET28a-$vapCB^{Ssonn}$
SH12	CTGGAGCTGATAAGGAGGCAAATAATGGAAACCACCGTATTTC	Construction of pET28a-$vapCB^{Ssonn}$
SH13	AGTGGTGGTGGTGGTGGTGCTCGAGTCAGAATGACTCCCTTTC	Construction of pET28a-$vapCB^{Ssonn}$

References

Datsenko KA, Wanner BL. One-step inactivation of chromosomal genes in *Escherichia coli* K-12 using PCR products. Proc Natl Acad Sci U S A. 2000 97:6640-5.

Guyer MS, Reed RE, Steitz T, Low KB. Identification of a sex-factor-affinity site in *E. coli* as gamma delta. Cold Spr. Harb. Symp. Quant. Biol. 1981 45:135-140

Guzman LM, Belin D, Carson MJ, Beckwith J. Tight regulation, modulation, and high-level expression by vectors containing the arabinose PBAD promoter. J Bacteriol. 1995 177:4121-30

Hanahan D. Studies on transformation with *Escherichia coli*. J Mol Biol. 1083 166:557-80.

Holt, K.E., Baker, S., Weill, F.X., Holmes, E.C., Kitchen, A., Yu, J., Sangal, V., Brown, D.J., Coia, J.E., Kim, D.W., Choi, S.Y., Kim, S.H., da Silveira, W.D., Pickard, D.J., Farrar, J.J., Parkhill, J., Dougan, G., and Thomson, N.R. (2012) *Shigella sonnei* genome sequencing and phylogenetic analysis indicate recent global dissemination from Europe. Nat Genet 44: 1056-1059.

Kopecko DJ, Washington O, Formal SB. Genetic and physical evidence for plasmid control of *Shigella sonnei* form I cell surface antigen. Infect Immun. 1980 29:207-14.

McVicker G, Tang CM. Deletion of toxin–antitoxin systems in the evolution of *Shigella sonnei* as a host-adapted pathogen. Nature Microbiology. 2016 2:16204.

McVicker G, Hollingshead S, Pilla G, Tang CM. Maintenance of the virulence plasmid in *Shigella flexneri* is influenced by Lon and two functional partitioning systems. Mol Microbiol. 2019 111:1355-66.

Miroux B, Walker JE. Over-production of proteins in *Escherichia coli*: mutant hosts that allow synthesis of some membrane proteins and globular proteins at high levels. J Mol Biol. 1996 260(3):289-298.

Sansonetti PJ, Kopecko DJ, Formal SB. *Shigella sonnei* plasmids: evidence that a large plasmid is necessary for virulence. Infect Immun. 1981 34:75-83.

Yanisch-Perron C, Vieira J, Messing J. Improved M13 phage cloning vectors and host strains: nucleotide sequences of the M13mp18 and pUC19 vectors. Gene. 1985 33:103-19.

Zychlinsky A, Prevost MC, Sansonetti PJ. *Shigella flexneri* induces apoptosis in infected macrophages. Nature. 1992 358:167-9.
